# Statistical optimization of light intensity and CO_2_ concentration for lipid production derived from attached cultivation of green microalga *Ettlia* sp.

**DOI:** 10.1038/s41598-018-33793-1

**Published:** 2018-10-18

**Authors:** Sungwhan Kim, Myounghoon Moon, Minsoo Kwak, Bongsoo Lee, Yong Keun Chang

**Affiliations:** 1 Department of Chemical and Biomolecular Engineering, KAIST, 291, Daehak-ro, Yuseong-gu, Daejeon 34141 Republic of Korea; 2 Advanced Biomass R&D Center, 291, Daehak-ro, Yuseong-gu, Daejeon 34141 Republic of Korea

**Keywords:** Biotechnology, Biodiesel

## Abstract

Attached cultivation systems have been receiving extensive attention as a breakthrough in microalgae cultivation technology. However, there is a lack of studies that emphasize precise optimization of important parameters in attached cultivation of microalgae. In this study, the effects of two major environmental parameters in photoautotrophic cultivation, light intensity and CO_2_ concentration, on the biomass and lipid surface productivity of *Ettlia* sp. YC001 were optimized by employing Response Surface Methodology (RSM) and validated experimentally. The optimum initial conditions for attached cultivation were use of seed from the late exponential phase (LE) and an inoculum surface density of 2.5 g/m^2^. By optimization, maximum biomass surface productivity of 28.0 ± 1.5 g/m^2^/day was achieved at 730 μE/m^2^/s with 8% CO_2_. The maximum lipid surface productivity was 4.2 ± 0.3 g/m^2^/day at 500 μE/m^2^/s with 7% CO_2_. Change of the fatty acid composition with respect to changes in environment parameters led to improvement of biodiesel quality at higher light intensity and higher CO_2_ concentration. Attached cultivation of *Ettlia* sp. YC001 has successfully produced biomass and lipids at a high production rate with relatively low light energy demand and high CO_2_ utilization.

## Introduction

Interest in photoautotrophically growing microalgae has rapidly increased in the past decade on the basis of its potential as a sustainable resource of energy, food, pharmaceuticals, and cosmetics, while mitigating CO_2_ concentration in the air and nitrogen and phosphorous concentration in wastewater. Despite its potential, the lack of economic competitiveness of microalgal biomass production hinders its commercialization, especially for products such as biofuel. Looking at the entire process, cultivation technology is considered one of the major reasons for this lack of economic competitiveness, where researchers encounter a number of significant obstacles that have not been resolved yet^1^. Ultimately, these obstacles facing conventional cultivation technology are fundamentally due to low biomass density (<1 g/L)^2^. Low density culture not only increases the risk of contamination by allowing contaminants to easily invade and dominate, but also increases the cost of harvesting and dewatering, which accounts for about 20% to 30% of the total cost of biomass production^3–5^. These major drawbacks must be resolved in large-scale production to make microalgae more economical^2,6,7^.

Unlike suspended cultivation systems, attached cultivation systems produce biomass in the form of a biofilm that has only about 70% water content (equivalent to 300 g/L biomass density), which significantly reduces cultivation volume, contamination risk, and cost of harvesting and dewatering^8^. A submerged biofilm cultivation system was developed based on the principle of natural formation of a biofilm on a bioreactor surface as the suspended culture becomes dense; however, a liquid layer flowing over the biofilm still acts as a barrier to the light and CO_2_ transfer, and does not prevent spread of contamination. On the other hand, another type of attached cultivation system, the Porous Substrate Bioreactor (PSBR), allows biofilm growth on a porous membrane that completely separates the biofilm from the liquid layer^9^. The liquid layer flows underneath the membrane through a so-called source layer. Water and nutrients from the source layer transfer through the porous membrane into the biofilm by diffusion and evaporative flux towards the surface of the biofilm. Compared to a submerged biofilm cultivation system, this principle of separation of the biofilm from the liquid layer allows for a much denser biofilm without the risk of detachment and spread of contaminants. Furthermore, it offers direct exposure of the biofilm to the ambient gas phase and the biofilm thereby can have better efficiency for light exposure and gas transfer^1,6,8,10^. As the PSBR-like system has potential to achieve extremely high biomass footprint productivity with very low operation cost, the system has recently been applied and evaluated with various microalgal species and environment parameters for various purposes^11–16^.

For photoautotrophically growing microalgae, especially in attached cultivation systems, light intensity and CO_2_ concentration are major environmental parameters that significantly affect biomass growth since these microalgae are directly exposed to the ambient air while using light energy to convert CO_2_ into biomass^16^. For this reason, fundamental studies on the effects of these parameters on biomass productivity in attached cultivation systems have been carried out, but extensive studies have not yet been reported. As light and CO_2_ work together in a complex manner, both parameters should be considered in combination^17^. However, conventional optimization studies have been conducted using a limited range of conditions, and may have led to unreliable results regarding optimization. It is thus important to optimize the light intensity and CO_2_ concentration for maximum biomass and lipid productivity by employing reliable and precise statistical tools such as Response Surface Methodology (RSM). This statistical optimization would allow for responses for a whole range of parameters with minimum experimental runs as well as prediction of the optimum biomass and lipid surface productivity for any combination of specific light intensity and CO_2_ concentration.

A green microalga species, *Ettlia* sp. YC001, has shown remarkable potential for biofuel production due to its large lipid content, but has been examined only in suspended cultivation systems^18^. *Ettlia* sp. YC001 is also known for producing extracellular polysaccharide substances (EPS), which makes it favorable for auto floc formation^19^. EPS is considered one of the major factors affecting biofilm formation, and thus *Ettlia* sp. YC001 has been recognized as a good candidate for attached cultivation. In this study, attached cultivation of *Ettlia* sp. YC001 was examined to determine the optimum initial conditions and optimized with respect to light intensity and CO_2_ concentration for maximum biofuel production.

## Results and Discussion

### Determination of initial conditions for attached cultivation of *Ettlia* sp. YC001

**Figure 1:**
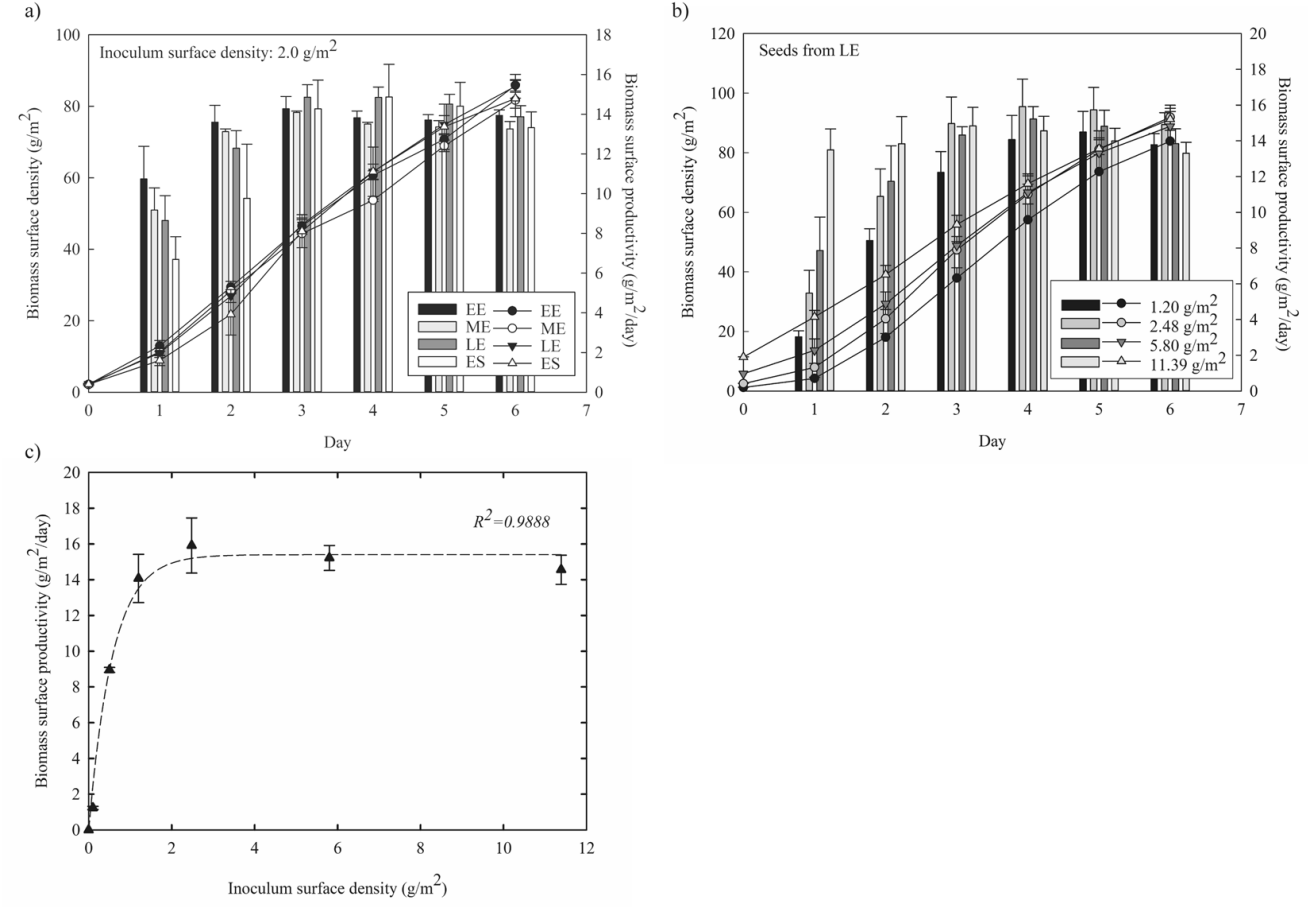
Effect of initial conditions on biomass production during attached cultivation of *Ettlia* sp. YC001. Initial conditions are (**a**) seed culture age of early exponential (EE, black bar, black circle), middle of exponential (ME, light gray bar, white cirlce), late exponential (LE, gray bar, black triangle), and early stationary (ES, white bar, white triangle) phases and (**b**) inoculum surface density of 1.20 g/m^2^ (black), 2.48 g/m^2^ (gray), 5.80 g/m^2^ (dark gray), and 11.39 g/m^2^ (light gray) for attached cultivation of *Ettlia* sp. YC001. Bar graph and line graph represent biomass surface productivity and biomass surface density, respectively. (**c**) Biomass surface productivity on day 4 (the maximum biomass surface productivity, black triangle) with respect to inoculum surface density is drawn along with regression line.

Prior to evaluating the effect of light intensity and CO_2_ concentration on attached cultivation of *Ettlia* sp. YC001, initial seed cultivation parameters such as seed age and inoculum density were examined. Seed from the early exponential phase (EE), middle exponential phase (ME), late exponential phase (LE), and early stationary phase (ES) was used as inoculum for attached cultivation, and the results of biomass production with respect to different seed ages are shown in Fig. 1a. Attached cultivation was conducted at 200 μE/m^2^/s with 2% CO_2_. Although seed from earlier phases showed higher biomass surface productivity in an earlier period of attached cultivation, seed from LE showed the highest biomass surface productivity from day 3 of attached cultivation to the rest of the cultivation period. Throughout the period, the maximum biomass surface productivity was achieved around day 4 and the seed from LE on day 4 also showed the highest biomass surface productivity. Thus, seed from LE was used for the remaining experiments in attached cultivation.

The effect of inoculum surface density was examined with seed from LE under a light intensity of 200 μE/m^2^/s with supplementary 2% CO_2_. Biomass surface productivity with different inoculum surface densities of 1.20, 2.48, 5.80, and 11.39 g/m^2^ is shown in Fig. 1b. On the first two days, the biomass surface productivity shows an incremental tendency as the inoculum surface density increases. However, from day 3 to the end of the cultivation period, the biomass surface productivity with an inoculum surface density of 2.48 g/m^2^ was the highest among the inoculum surface densities. The biomass surface productivity on day 4 with respect to the inoculum surface density and its respective regression line is shown in Fig. 1c. The regression line drawn in this figure has an adjusted *R*^2^ value of 98.7%, which is high enough to indicate the model is adequate. A sharp increase in the biomass surface productivity is shown from an inoculum surface density of 0.1 to approximately 2.5 g/m^2^. It then levels off at a biomass surface productivity of 16 g/m^2^/day. A similar tendency of saturation at optimum inoculum surface density has been reported in a number of studies. One study reported that attached cultivation of *S. platensis* in a similar system showed the maximum biomass productivity on both day 1 and day 3 with an inoculum surface density of around 7 to 11 g/m^2^, and then decreased with an increase in inoculum surface density^20^. Another study reported that the optimum inoculum surface density of 3 to 5 g/m^2^ with *P. Pseudochlorococcum* was observed based on the biomass productivity on day 6^21^. Based on the findings presented in this section, seed from LE with an inoculum surface density of 2.5 g/m^2^ was chosen as the optimum initial conditions for the attached cultivation and used for the remaining experiments.

### Effect of light intensity and CO_2_ concentration on attached growth of *Ettlia* sp. YC001

**Figure 2:**
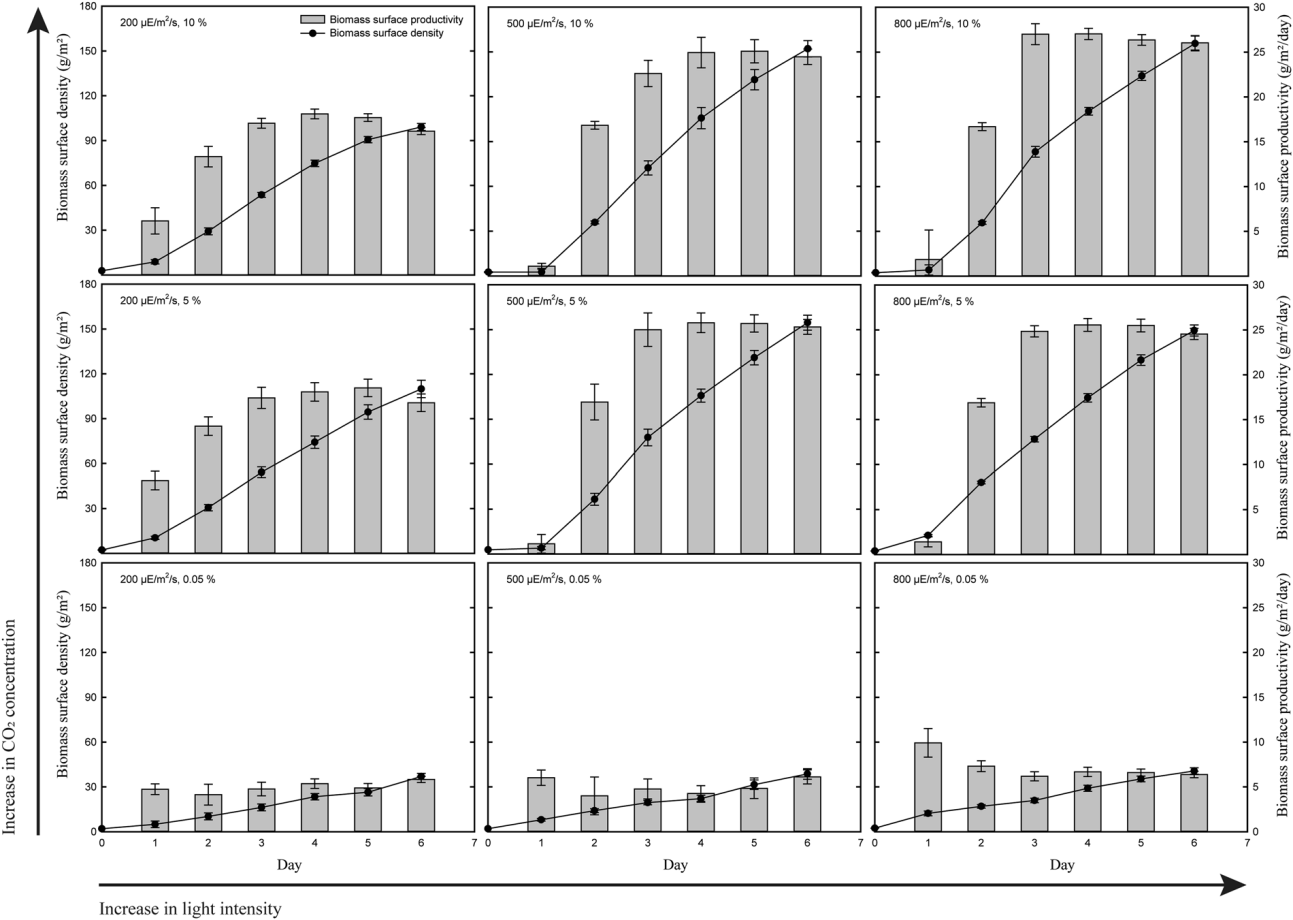
Effect of light intensity and CO_2_ concentration on biomass production. Each figure represents biomass production during attached cultivation of *Ettlia* sp. YC001 under different light intensity and CO_2_ concentration. Figures from left to right, light intensity increases from 200 to 500 to 800 μE/m^2^/s. From bottom to top, CO2 concentration increases from ambient level (0.05%) to 5% to 10%. Biomass surface densities (line) and biomass surface productivities (bar) are shown daily throughout the entire cultivation period. All figures are in same scale.

The biomass surface density and the productivity graphs at different light intensities and CO_2_ concentrations are shown in Fig. 2. Biomass growth of *Ettlia* sp. YC001 was measured daily in order to determine if the growth behavior varies with respect to different light intensity and CO_2_ concentration. With ambient air (0.05% of CO_2_), attached cultivation of *Ettlia* sp. YC001 showed poor biomass production regardless of light intensity. The biomass surface productivity averaged around 4 to 6 g/m^2^/day throughout the whole period of cultivation. The final biomass surface density on day 6 only slightly increased (from 37 to 40 g/m^2^) at light intensity of 200 to 800 μE/m^2^/s. Since *Ettlia* sp. YC001 was originally isolated on the basis of showing a high growth rate and high lipid productivity under a high concentration of CO_2_, supplement of enough CO_2_ appeared to be essential for a healthy biomass production^18^. With supplement of 5% and 10% CO_2_, biomass growth increased dramatically even at a low light intensity of 200 μE/m^2^/s. There was also an increase in biomass growth as the light intensity was increased from 200 µE/m^2^/s to the higher intensities. Biomass surface productivity at light intensities of 500 and 800 µE/m^2^/s, however, showed a lag phase for the first 24 hours. Despite showing a lag phase, higher biomass surface productivity was shown from day 2 at a higher light intensity. The maximum biomass surface productivity was achieved on day 4 and then slowly decreased thereafter. Thus, biomass surface productivity on day 4 was chosen to be optimized.

### Optimization of biomass surface productivity by employing RSM

Optimization of light intensity and CO_2_ concentration to achieve the maximum biomass surface productivity of *Ettlia* sp. YC001 was conducted by employing Central Composite Face-centered (CCF) design and RSM. The CCF design was selected because it allows the experiment conditions to be kept within the desired range of independent variables. The independent variables, light intensity and CO_2_ concentration, were selected corresponding to the coded levels of −1, 0, and 1, which are 200 μE/m^2^/s, 500, μE/m^2^/s and 800 μE/m^2^/s for light intensity, and ambient air (0.05%), 5%, and 10% for CO_2_ concentration, respectively (Supplementary Table S1). A total of 29 experiments, including 5 center points and 8 triplicated experiments, were conducted based on the CCF design. Observed and predicted responses of biomass surface productivity of each point are shown in Supplementary Table S2. Predicted responses were calculated based on the equation with a regression coefficient for each term from the quadratic model. The regression equation in terms of coded factors was defined as equation (1):

1 $${P}_{B}=24.44+2.41\cdot {\rm A}+8.80\cdot {\rm B}+1.75\cdot A\cdot B-2.21\cdot {{\rm A}}^{2}-8.65\cdot {{\rm B}}^{2}$$

where *P*_*B*_ is the biomass surface productivity and A and B are coded levels of the independent variables, light intensity and CO_2_ concentration, respectively. Equation (1) was then recast in terms of the actual values of independent variables, as shown in equation (2):

2 $${P}_{B}=-\,0.47+0.03\cdot Light+4.70\cdot C{O}_{2}+1.17\times {10}^{-3}\cdot Light\cdot C{O}_{2}-2.45\times {10}^{-5}\cdot Ligh{t}^{2}-0.35\cdot C{{O}_{2}}^{2}$$

Significance and adequacy of the developed quadratic model were statistically evaluated using ANOVA and the results are shown in Table 1. As the p-values of individual terms in the model along with the model itself were less than 0.05, they have a significant effect on the response, and thus the model is significantly supported. The determination coefficient (*R*^2^) of the quadratic model was 0.9498, indicating a high correlation between independent and dependent variables. The adjusted R^2^ (Adj-*R*^2^) value was determined by adjusting R^2^ for the number of terms in the model, and the predicted *R*^2^ (Pred-*R*^2^) value is a measure of the amount of variation in new data explained by the model. The difference between Adj-*R*^2^ and Pred-*R*^2^ was within a reasonable range (<0.2). The adequate precision value, a signal to noise ratio, was greater than 4, indicating adequacy of the model discrimination.

**Table 1:** ANOVA of the quadratic model for biomass surface productivity of *Ettlia* sp. YC001.

Source	Sum of squares	degree of freedom	Mean square	F-value	p-value
Model	2110.29	5	422.06	91.58	<0.0001
A-Light	104.43	1	104.43	22.66	<0.0001
B-CO2	1393.88	1	1393.88	302.44	<0.0001
AB	36.78	1	36.78	7.98	0.0099
A^2^	32.74	1	32.74	7.10	0.0141
B^2^	503.22	1	503.22	109.19	<0.0001
Residual	101.39	22	1.38		
Lack of Fit	72.54	11	6.59	2.51	0.0708
Pure Error	28.86	11	2.62		
R^2^	95.42%				
Adj-R^2^	94.37%				
Pred-R^2^	92.09%				
Adeq. Precision	21.604				

**Figure 3:**
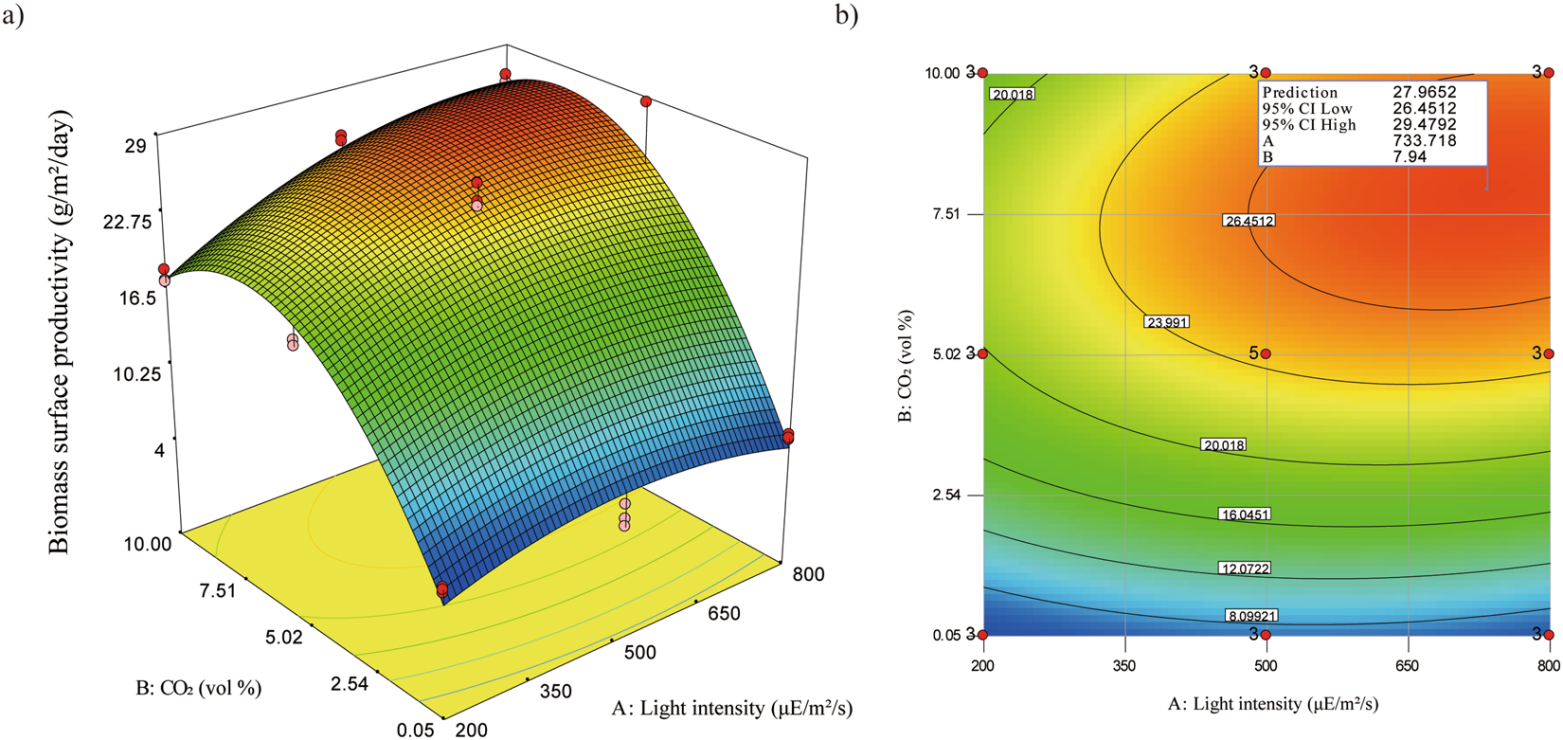
Response surface of biomass surface productivity in attached cultivation of *Ettlia* sp. YC001. Both of (**a**) three-dimensional plot and (**b**) contour plot are drawn based on the predicted biomass surface productivity on day 4 by the quadratic model developed in this study. In contour plot, an optimum point is indicated with information of the maximum biomass surface productivity, 95% confidence interval low and high, and conditions for the optimum point.

Based on the predicted responses of the quadratic model, the response surface was drawn as a 3D surface and a contour plot, shown in Fig. 3a,b. Based on the model, the maximum biomass surface productivity was 28.0 ± 1.5 g/m^2^/day at around 730 μE/m^2^/s with 8% CO_2_ concentration. The 95% confidence interval (CI) of the maximum biomass surface productivity ranged from 26.5 g/m^2^/day to 29.5 g/m^2^/day. The boundary of the low 95% CI of the maximum biomass surface productivity is also shown in the contour plot in Fig. 3b. In this study, the low 95% CI boundary was interpreted as the optimum range for the maximum biomass surface productivity of *Ettlia* sp.YC001. At the low 95% CI boundary, coordination with the minimum light intensity and the minimum CO_2_ concentration were 490 μE/m^2^/s with 7.5% and 6% with 650 uE/m^2^/s, respectively. The information about the minimum conditions for the maximum biomass surface productivity is useful since it determines how efficient the system can be.

Two studies involving similar systems that achieved higher biomass surface productivity of 31.2 g/m^2^/day with *Halochlorella rubescens*^17^ and 29 g/m^2^/day with *Scenedesmus vacuolatus*^22^ grown in twin-layer PBRs have been reported. The growth conditions for the respective studies were light intensity and CO_2_ concentration of 1023 μE/m^2^/s and 3%, and 600 μE/m^2^/s and 2%, both with a light/dark cycle of 14/10 hours. In order to compare the biomass productivity in a similar system but with different light intensity, which is a major source of energy input, biomass productivity can be normalized by dividing it by the amount of light energy required. This concept is discussed in other studies as well, where it has been described as photosynthetic efficiency, solar energy conversion efficiency, light utilization efficiency, or photoefficiency^17,23–25^. The aforementioned factor is considered important to evaluate the growth performance of photoautotrophic microalgal species more precisely. Thus, biomass yield over light energy input is calculated and compared with the results obtained in other studies in order to measure its competitiveness with similar systems. Carbone, D. A., *et al*.^22^ calculated the photosynthetic efficiency based on the equation reported by Schultze, L. K., *et al*.^17^, which is expressed in terms of biomass produced in grams per moles of photons exposed in unit of Einstein (E), and compared the results with those in other studies involving similar systems^17,22^. The photosynthetic efficiency of the two highest biomass surface productivities of *H. rubescens* and *S. vacuolatus* previously mentioned were 0.6 and 0.95 g/E, respectively. Although *H. rubescens* achieved the highest biomass surface productivity, the required light intensity was higher than that required for *S. vacuolatus*, and thus the photosynthetic efficiency was smaller. Based on Schultze’s calculation, the photosynthetic efficiency of the maximum biomass surface productivity at the optimum point achieved in this study was about 0.4. Meanwhile, the photosynthetic efficiency of the attached growth of *Ettlia* sp. YC001 can be improved with the maximum biomass productivity at the minimum light intensity of the optimum range, which is around 0.7 g/E. Furthermore, *Ettlia* sp. YC001 was grown under continuous light, which perhaps resulted in lower light use efficiency, and thus a proper light/dark cycle strategy may increase its photosynthetic efficiency even further.

### Effect of light intensity and CO_2_ concentration on lipid content

**Figure 4:**
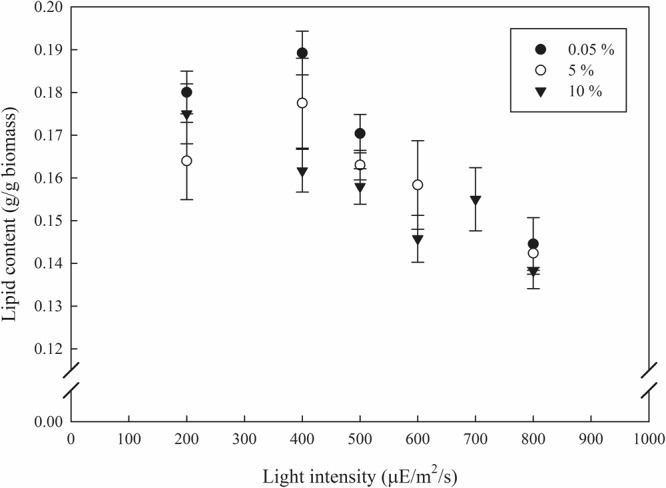
Effect of light intensity on lipid content of *Ettlia* sp. YC001 under different CO_2_ concentration. At each light intensity (except 600 μE/m^2^/s and 700 μE/m^2^/s), ambient air (dark circle), 5% (empty circle), and 10% (dark inverted triangle) were tested.

Light intensity and CO_2_ concentration not only affect biomass growth, but also affect the biomass biochemical composition such as lipids^4,26^. In general, high light intensity acts as a stress stimulating cells to induce TAG production in order to protect themselves from the excess light energy^26–28^. For an attached cultivation system, however, little study has been conducted regarding the effects of light intensity and CO_2_ concentration on lipid contents. A study employing a submerged biofilm cultivation system reported the effect of light intensity on lipid content and showed no increase at light intensities from 150 to 600 μmol/m^2^/s, although there was a sharp increase at 50 μmol/m^2^/s^29^. The authors explained the aforementioned phenomenon, a so called muted effect, was due to a shading effect caused by the upper layer of the biofilm^29^. In other words, only the top layer of the biofilm exposed at high light intensity accumulates more TAG to protect it from the excess energy input, while the bottom layer of the biofilm experiences much lower light intensity and thus no further TAG production is activated. In this study, lipid content showed a decreasing tendency from about 19% to 14% with an increase in light intensity, as shown in Fig. 4. This phenomenon is presumably attributable to the muted effect explained by Schnurr, P. J., *et al*.^4^. Under stressful condition with high light intensity, supplement of CO_2_ can contribute to accumulate larger amount of TAG than where there is no supplement of CO_2_^27^. For instance, a previous study demonstrated that the supplement of CO_2_ under nitrogen depletion conditions resulted in an increase in TAG whereas there was no TAG accumulation with ambient air^30^.

In the case of a biofilm with supplement of CO_2_, owing to its heterogeneity, only the top layer of the biofilm experiences stress under nutrient depletion with consistent exposure to high light intensity, and may result in increased TAG to store carbon, meanwhile, the bottom layer of the biofilm experiences no stress resulting in accumulation of starch to store carbon. For instance, an attached cultivation study reported changes in lipid and TAG contents of *Scenedesmus dimorphus* under various concentration of CO_2_ concentration^16^. Lipid and TAG contents increased under CO_2_ concentration from 0.04% to about 0.2%. Under higher CO2 concentration range, lipid content maintained, however, TAG content slightly decreased as CO_2_ concentration further increased up to 10%. Although tendency was found regarding the effect of CO_2_ concentration on lipid and TAG content, entire experiment was conducted under single and low light intensity, thus, more information with range of light intensity is needed^16^. In this study, although lipid content varied little with CO_2_ concentrations, there is a slight decreasing tendency with an increase in CO_2_ concentration from ambient air to 10%. This phenomenon was observed larger with lower light intensity. It is suggested that at higher light intensity, above 400 μE/m^2^/s in this study, light intensity becomes a dominant factor over CO_2_ concentration. It is well reported that the high light intensity stimulates microalgae to utilize more carbon to transform into neutral lipid^26–28^. In this study, light intensity acted as a limiting factor under 400 μE/m^2^/s and lower light intensity so that the effect of CO_2_ concentration on lipid content was larger, however, higher light intensity was enough to utilize all the carbon microalgae can uptake even at low CO_2_ concentration, therefore, not much difference in lipid content among with variation in CO_2_ concentration was observed.

In general, as the concentration of CO_2_ increases from 0.05%, biomass growth is significantly enhanced, meaning biofilm is thickened. As the biomass increases, a portion of the top layer experiencing high light stress appears to remain unchanged while the amount of biomass in the bottom layer increases, where negligible lipid accumulation occurs. Thus, under favorable conditions for biofilm growth, higher light intensity with supplement of CO_2_, the lipid content of the entire biofilm decreases due to an increase in amount of low-lipid biomass in the bottom layer.

### Effect of light intensity and CO_2_ concentration on fatty acid composition and biodiesel quality

The quality of produced lipids is equally important as their quantity in terms of serving as a source of transportation fuel. Along with fatty acid compositions, key parameters for the biodiesel quality evaluation are determined and listed in Table 2.

**Table 2:** Fatty acid composition and biodiesel quality analysis.

CO_2_(vol %)	Light (µE/m^2^/s)	Total FAME (wt. %)	Fatty acid composition (wt%)											CN ^a^	IV^b^	DU^c^	LCSF^d^	CFPP^e^ (°C)
			*C10:0*	*C14:0*	*C16:0*	*C16:1*	*C18:0*	*C18:1n9*	*C18:2n6*	*C18:3n3*	SFA	MUFA	PUFA					
0.05	200	18.0%	3.1	—	15.6	3.1	3.2	32.7	14.2	22.7	21.8	35.8	36.9	48.9	120.4	109.6	3.1	−6.6
	500	18.9%	2.8	—	15.3	2.9	3.1	33.2	13.4	24.4	21.2	36.1	37.8	48.4	123.7	111.6	3.1	−6.8
	800	12.1%	4.0	—	17.1	2.2	2.5	27.0	12.9	30.6	23.6	29.2	43.5	47.0	133.6	116.2	3.0	−7.1
5	200	17.0%	1.1	—	18.9	4.0	3.1	35.5	16.1	14.3	23.2	39.5	30.4	51.5	104.3	100.3	3.4	−5.7
	500	16.1%	2.6	—	16.6	2.6	2.4	33.5	14.9	15.3	21.7	36.1	30.3	51.3	101.8	96.7	2.9	−7.4
	800	14.2%	3.5	—	15.8	2.1	2.7	29.6	15.5	19.1	22.0	31.7	34.6	50.1	109.1	100.9	2.9	−7.3
10	200	17.5%	1.1	1.3	17.9	3.1	2.2	29.6	15.2	14.1	22.6	32.7	29.3	52.3	96.0	91.4	2.9	−7.4
	500	15.8%	2.0	0.5	18.0	2.7	2.5	30.7	12.7	18.3	22.9	33.4	31.0	51.3	103.5	95.4	3.0	−6.9
	800	13.8%	4.8	1.0	16.8	1.9	2.2	25.0	13.6	20.4	24.9	26.9	34.0	50.8	105.0	95.0	2.8	−7.7

^a^CN = 62.2 + 0.017 L + 0.074 M + 0.115 P + 0.177 S _ 0.103 O _ 0.279LI _ 0.366LL, where L, M, P, S, O, Li and LL are the weight percentages of methyl esters, as follows: C12:0, C14:0, C16:0, C18:0, C18:1, C18:2, and C18:3, respectively^31^.

^b^IV was determined according to the European standard method (EN 14214).

^c^DU = 1 (monounsaturated Cn: 1, wt.%) + 2 (polyunsaturated Cn: 2, 3, wt.%)^34^.

^d^LCFA = 0.1 C16 (wt.%) + 0.5 C18 (wt.%) + 1 C20 (wt.%) + 1.5 C22 (wt.%) + 2 C24 (wt.%)^34^.

^e^CFPP = 3.0147 LSCF = 16:477^34^.

Among algae, the most common synthesized fatty acids have chain length ranging from C16 to C18^27^. C16 to C18 account for more than 90% of total fatty acid (TFA) in attached grown *Ettlia* sp. YC001 together with a small portion of medium chain length fatty acids such as C10 and C14. The composition of fatty acids found in this study is consistent with the results from the original report of *Ettlia* sp. YC001^18^.

With an increase in light intensity, the composition of capric acid (C10:0) and palmitic acid (C16:0), along with that of linolenic acids (C18:3n3) increased, whereas stearic acid (C18:0), palmitoleic acid (C16:1), oleic (C18:1), linoleic (C18:2), and alpha linolenic acids (C18:3) decreased. In summary, while TFA content decreased with respect to an increase in light intensity, the proportion of PUFA and C10:0 increased whereas major SFA (C16:0, C18:0) and MUFA decreased. Interpretation of this phenomenon is complicated since the biofilm is not homogenous, and thus a different biochemical composition with respect to the depth of the biofilm is expected. In terms of its effect on fatty acid composition, high light intensity is generally known to reduce the composition of PUFA while causing an increase in SFA and MUFA due to synthesis of neutral lipids^27^. In this study, however, as indicated by the decreasing tendency of TFA, the composition of PUFA increases due to an increase in the amount of biomass of the bottom layer of the biofilm where more photosynthetic activity occurs, while major SFA and MUFA decreased due to a decrease in the portion of the top layer biomass where TAG is synthesized.

In order to further evaluate the quality of produced lipids, the following key parameters were studied to determine biodiesel quality: cetane number (CN), iodine value (IV), oxidation stability, and cold filter plugging point (CFPP). These factors determine the adequacy of biodiesel, which depends largely on the fatty acid compositions^31,32^. The CN, one of the most significant parameters regarding ignition delay time, decreased with an increase in light intensity, but increased with supplement of CO_2_. It is known that the CN increases with an increase in chain length, but decreases with an increase in the degree of unsaturation^31^. In this study, C18:3, the largest portion of TFA, increased with an increase in light intensity, which led to a decrease in the CN with an increase in light intensity. With respect to supplement of CO_2_, there is a decrease in the portion of C18:3, and therefore there is an increase in the CN associated with an increase in CO_2_ concentration. This result indicates that cultivation with supplement of CO_2_ rather than with ambient air has a considerable impact on increasing the CN, while an increase in light intensity only slightly decreases the CN. Overall, the CN in all conditions satisfied both European (EN 14214, >51) and American (ASTM D6751, >47) standards^33^. The oxidation stability decreases with an increase in the content of PUFA^34^. In this study, the DU decreased with an increase in CO_2_ concentration, whereas it slightly increased with an increase in light intensity. Thus, lipids from the biomass grown under a higher concentration of CO_2_ are more stable. The IV has a meaning similar to that of DU and accordingly shows the same tendency. The calculated CFPP indicates the lowest temperature before filter plugging occurs. This value has a positive correlation with compositions of C16:0 and C18:0. The CFPP increased with respect to both an increase in light intensity and an increase in CO_2_ concentration. In summary, the CN and the CFPP ranged from around 47 to 53 and −6.6 to −7.7 °C, respectively. Light intensity had a negative impact on the CN, but a weak and positive impact on the CFPP. Supplementary CO_2_ had a positive impact on both the CN and the CFPP. Thus, a weak but increasing tendency of biodiesel quality was observed with decreasing light intensity and increasing CO2 concentration within the range covered in this study.

### Optimization for lipid surface productivity by employing RSM

Based on the maximum biomass surface productivity and its lipid content, the maximum lipid surface productivity with respect to light intensity and CO_2_ concentration was found by employing RSM. A quadratic model was developed and predicted responses were calculated based on the equation with the regression coefficient for each term from the quadratic model. The regression equations in terms of coded and actual factors were defined as equations (3) and (4):

3 $${P}_{Lipid}=3.93+0.055\cdot {\rm A}+1.12\cdot {\rm B}-0.034\cdot A\cdot B-0.66\cdot {{\rm A}}^{2}-1.30\cdot {{\rm B}}^{2}$$

4 $$\begin{array}{c}{P}_{Lipid}=-\,0.521+7.67\times {10}^{-3}\cdot Light+0.77\cdot C{O}_{2}-2.27\times {10}^{-5}\cdot Light\\ \,\,\,\,\,\,\,\cdot C{O}_{2}-7.37\times {10}^{-6}\cdot Ligh{t}^{2}-0.05\cdot C{{O}_{2}}^{2}\end{array}$$

where *P*_*Lipid*_ is lipid surface productivity, and A and B are coded levels of the independent variables, light intensity and CO_2_ concentration, respectively. The results of ANOVA to evaluate the statistical significance and adequacy of the developed model are shown in Table 3. The p-value of the developed model is less than 0.0001, indicating only 5% noise, and thus the model is significantly supported, although there are two terms, A and AB, that have a p-value greater than 0.05. The terms A, coded level of light intensity, and AB, coded level of both light intensity and CO_2_ concentration, have large p-values, presumably due to the opposite tendencies of biomass surface productivity and lipid content with respect to light intensity. This conflict might lead to an unclear interpretation of the effect of light intensity and the combination of light intensity and CO_2_ concentration, and thus may lead to those model terms being insignificant. However, the p-value of the term “Lack of fit” is greater than 0.05, indicating the model is fit and thus adequate to be used. In addition, the adj-R^2^ value and pred-R^2^ value have a difference of less than 0.2, indicating the adequacy of the model.

**Table 3:** ANOVA of the quadratic model for lipid surface productivity of *Ettlia* sp. YC001.

Source	Sum of squares	degree of freedom	Mean square	F-value	p-value
Model	38.51	5	7.70	52.91	<0.0001
A-Light	0.06	1	0.05	0.38	0.5458
B-CO2	22.43	1	22.43	154.06	<0.0001
AB	0.01	1	0.01	0.09	0.7609
A^2^	2.96	1	2.96	20.31	0.0002
B^2^	11.43	1	11.43	78.49	<0.0001
Residual	3.20	22	0.15		
Lack of Fit	2.12	11	0.19	1.95	0.1416
Pure Error	1.09	11	0.10		
R-Squared	92.32%				
Adj R-Squared	90.58%				
Pred- R^2^	87.01%				
Adeq. Precision	17.08				

**Figure 5:**
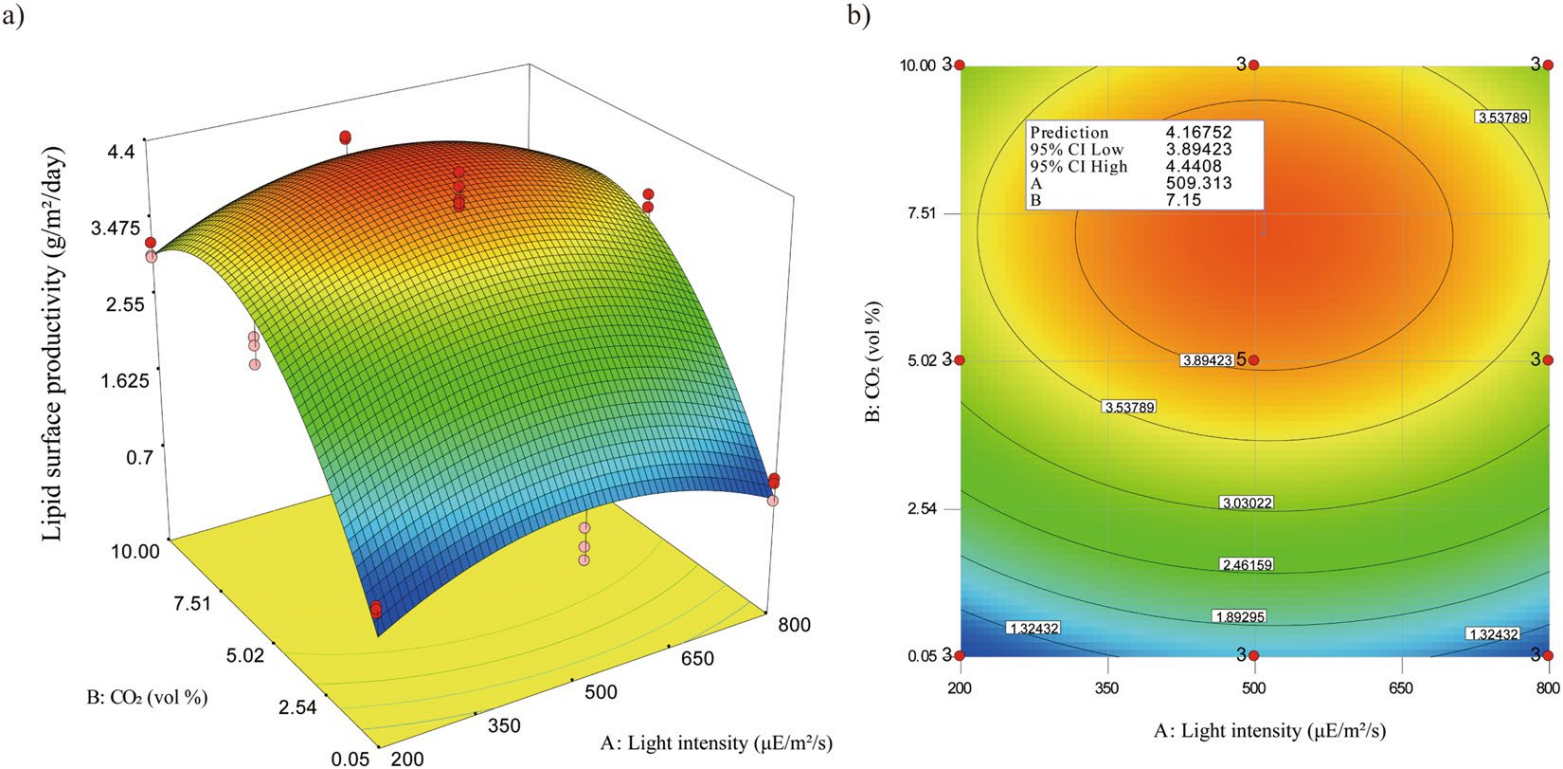
Response surface of lipid surface productivity in attached cultivation of *Ettlia* sp. YC001. Both of (**a**) three-dimensional plot and (**b**) contour plot are drawn based on the predicted lipid surface productivity on day 6 by the quadratic model developed in this study. In contour plot, an optimum point is indicated with information of the maximum biomass surface productivity, 95% confidence interval low and high, and conditions for the optimum point.

The predicted responses based on the designed quadratic model were developed and plotted as a 3D surface and contour plot, as shown in Fig. 5a,b. Because the lipid content of the biomass increased with a decrease in light intensity and CO_2_ concentration, the location of the optimum point for the maximum lipid surface productivity shifted from that for the maximum biomass surface productivity towards lower light intensity and CO_2_ concentration. As a result, the maximum lipid surface productivity was 4.2 ± 0.3 g/m^2^/day at about 500 µE/m^2^/s with 7% CO_2_ concentration. To the best of our knowledge, reported lipid productivities in similar cultivation systems are around 1 to 3 g/m^2^/day^11,35,36^. Although lipid content of attached grown *Ettlia* sp. YC001 is relatively low compared to other reported oleaginous species, the maximum lipid surface productivity achieved in this study is considered one of the highest lipid surface productivities reported by far due to its high biomass surface productivity. Development of a strategy to induce greater lipid accumulation in the bottom layer of the biofilm may enhance lipid surface productivity of attached cultivation *Ettlia* sp. YC001 even further.

### Validation of the model

**Figure 6:**
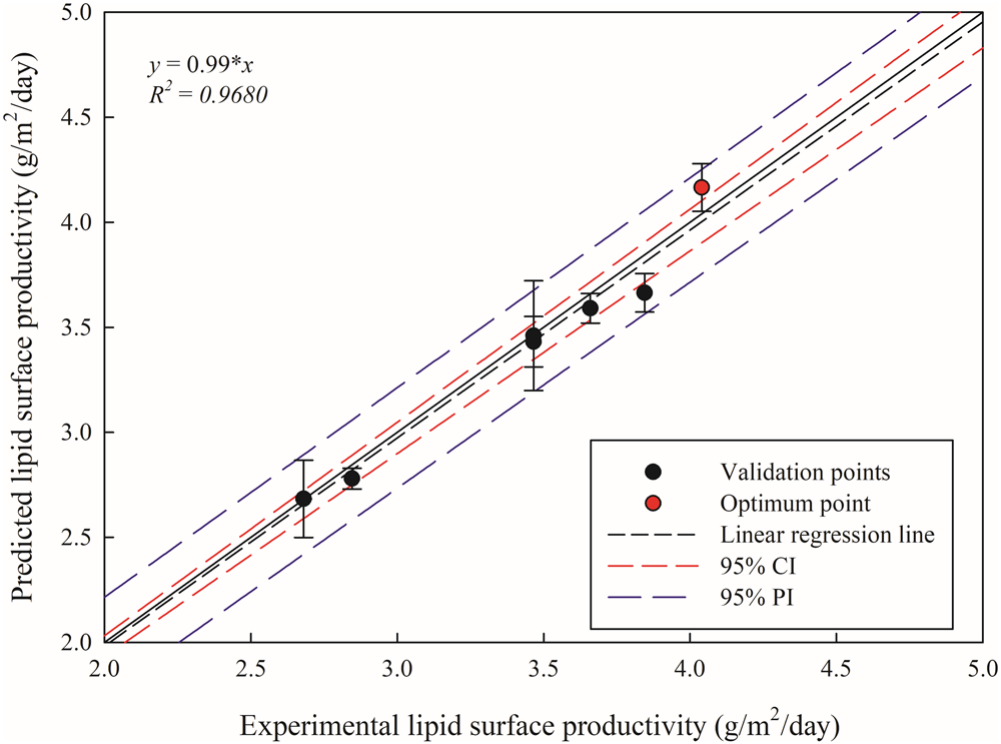
Correlation between the experimental and the predicted lipid surface productivities. Six different locations (black circle) besides designed points, along with an optimum point (red circle), were chosen to validate the quadratic model developed in this study. Linear regression line (black dashed line) with 95% confidence interval (red dashed line) and 95% predicted interval (blue dashed line) are drawn for adequacy of the validation.

Validation of the model was conducted with six different locations of the lipid surface productivity within the tested range of light intensity and CO_2_ concentration along with the optimum point. Locations for validation are at light intensity of 300 µE/m^2^/s and 700 µE/m^2^/s each with supplement of 2.5%, 5%, and 10% CO_2_. Both the predicted and experimental values of lipid surface productivity at validation points along with the optimum point are shown in Fig. 6. The correlation between the predicted and experimental data was linear with a linear regression line of y = 0.99 · x with *R*^2^ = 0.9680. Considering error bars, all data are within 95% CI. The experimental value of the maximum lipid surface productivity was 4.0 ± 0.1 g/m^2^/day, which lies within 95% CI of the predicted value of the maximum lipid surface productivity of 4.2 ± 0.3 g/m^2^/day.

## Conclusions

Viability of attached cultivation of *Ettlia* sp. YC001 was verified, along with optimization of initial conditions and environmental parameters for attached cultivation. The optimum conditions for the maximum biomass and lipid surface productivity were 28.0 ± 1.5 g/m^2^/day at 730 μE/m^2^/s with 8% and 4.2 ± 0.3 g/m^2^/day at 500 µE/m^2^/s with 7% of CO_2_ concentration, respectively. These results show that attached cultivation of *Ettlia* sp. YC001 successfully produced biomass and lipids at a high production rate with relatively low light energy demand and high CO_2_ utilization. Attaining a sound understanding of the variation of the biochemical composition of the biofilm layer by layer is suggested in order to optimize lipid accumulation, which may further enhance biofuel production of attached cultivation of *Ettlia* sp. YC001.

## Methods

### Microalgal strain, medium, and inoculum preparation

The freshwater green microalgal species, *Ettlia* sp. YC001 (KCTC 12109BP), was obtained from the Korean Collection for Type Cultures (KCTC) at the Korea Research Institute of Bioscience and Biotechnology (KRIBB). The *Ettlia* sp. YC001 was maintained on a TAP agar plate for long storage, and suspended in BG11 media and photoautotrophically cultivated when seed was needed for the attached cultivation. Seed culture was prepared in an autoclaved 500 ml bottle with an air inlet (working volume was 400 ml). Continuous light intensity of 200 μE/m^2^/s with supplementary 2% CO_2_ was used for all seed preparation. BG11 was used as a medium in both suspended and attached cultivation, and it consists of 1.5 g/L of NaNO_3_, 0.23 mM K_2_HPO_4_, 0.3 mM MgSO_4_·7H_2_O, 0.24 mM CaCl_2_·2H_2_O, 0.031 mM Citric Acid·H_2_O, 0.021 mM Ferric Ammonium Citrate, 0.0027 mM Na_2_EDTA·2H_2_O, 0.19 mM Na_2_CO_3_, and 1 ml/L of BG-11 trace metals solution of 46 mM H_3_BO_3_, 9 mM MnCl_2_·4H_2_O, 0.77 mM ZnSO_4_·7H_2_O, 1.6 mM Na_2_MoO_4_·2H_2_O, 0.3 mM CuSO_4_·5H_2_O, and 0.17 mM Co(NO_3_)_2_·6H_2_O. The pH of the medium was adjusted in a range of 7 to 7.5 using 1 N of HCl and NaOH prior to all experiments including inoculum preparation.

### Attached cultivation system

**Figure 7:**
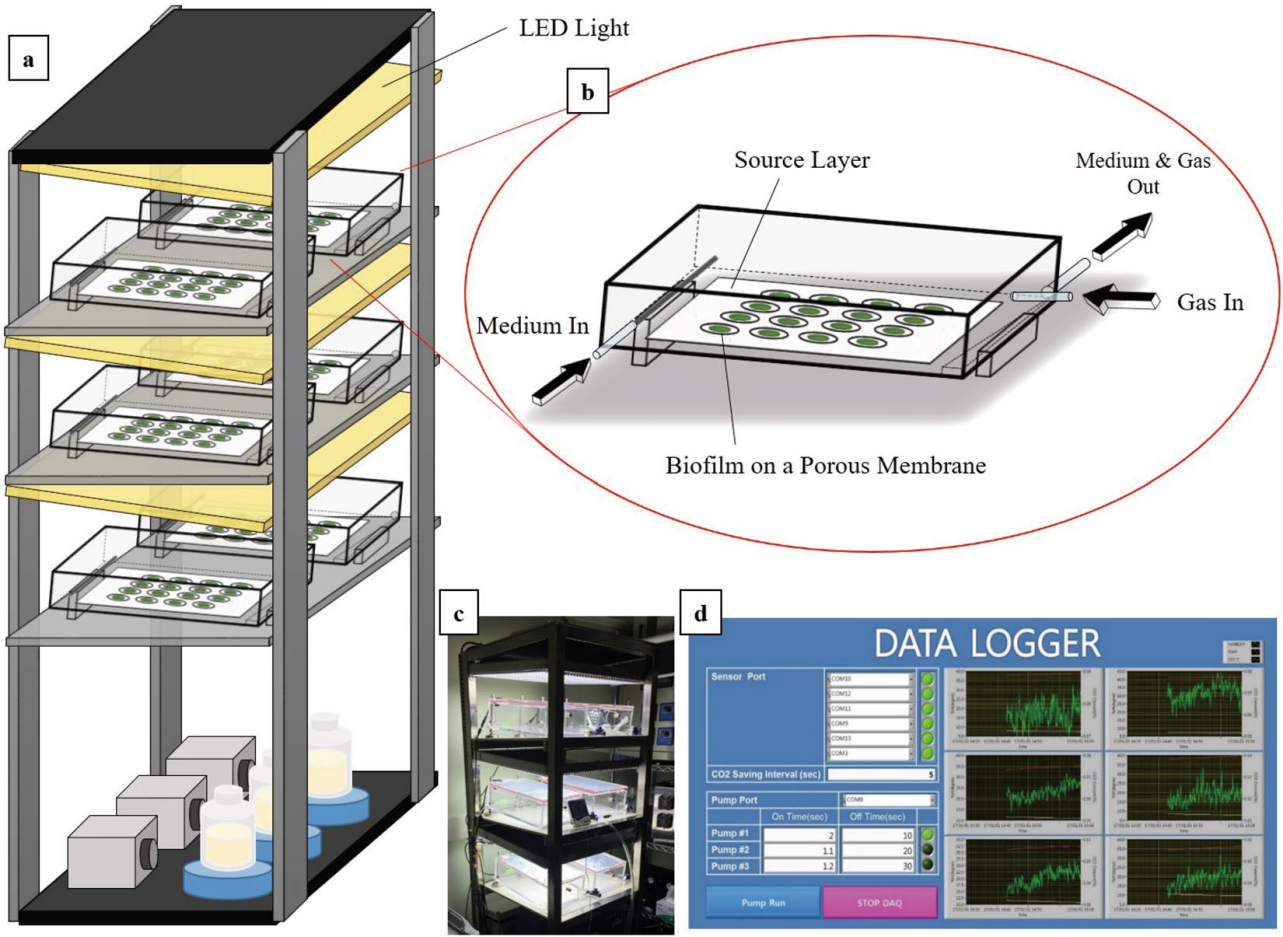
Schematic diagram of the attached cultivation system. (**a**) Image of the entire attached cultivation system, (**b**) detail image of each bioreactor, (**c**) actual photo of the system, (**d**) and real-time data logging program for temperature, humidity, and CO_2_ concentration inside of each bioreactor.

The attached cultivation system used in this study is shown in Fig. 7a,c. The bioreactor was developed based on the principle of PSBR, which consists of a porous membrane substrate, a source layer, a medium supplying stainless steel tube, a gas inlet, and a liquid outlet (Fig. 7b). Biomass was inoculated using a vacuum pump on a porous membrane substrate (nitrocellulose membrane, 47 mm, the pore size of 0.45 um, Millipore) with an inoculum size of 30 mm diameter. The inoculated porous membrane was then placed on the source layer in the bioreactor. The medium was pumped by a peristaltic pump and supplied on the source layer through the medium supplying stainless steel tube. To ensure enough nutrients and moisture are supplied, 1.5 ml of medium for every 2.5 min was supplied for all experiments. The medium supplied on the source layer was then transferred to the biofilm through the porous membrane substrate. Each bioreactor was tilted to ensure the medium flowed from the top to bottom of the bioreactor. The excess medium was then naturally removed by gravity through the liquid outlet located on the bottom of the bioreactor. Gas (air or air with CO_2_) was supplied at 700 ml/min through the air filter installed on the gas inlet, which was enough to keep inside of the reactor at constant level of desired CO_2_ concentration during entire cultivation period. Temperature and CO_2_ volumetric concentration throughout the system were monitored and recorded periodically by sensors (COZIRTM Wide Range Sensor, GSS Ltd) installed on the top side of each bioreactor (Fig. 7d). The temperature for all experiments was kept at around 27 °C ± 1 °C. The bioreactors were installed under the manufactured LED panel (Cool white, 8000 K), which was also tilted parallel to the bioreactor. Fans were installed on both the front and rear side of the LED panel to prevent the temperature of the bioreactor from rising due to the heat generated by the LED panel.

### Growth analysis

The conventional way to measure the growth of a biofilm is to sacrifice a sample periodically to weight its dry biomass (W_DB_). For this method it must be assumed that all samples grow equally under the same conditions. Thus, the conventional growth analysis method would make the experiment not only inaccurate, but also time consuming and laborious since many replicated samples are required in order to draw a single growth curve under one condition. In this study, a new growth analysis method, wet weight measurement, was developed and employed so that no sacrifice of samples was required. The wet weight measurement method predicts W_DB_ of the sample by developing a correlation between W_DB_ and the corresponding weight of the wet biomass (W_WB_). In order to measure W_WB_, the weight of wet sample (W_WS_) was measured using an analytical balance under a humid condition after removing free water via a vacuum pump. And then the weight of wet filter (W_WF_) was measured after biomass was harvested. W_WB_ was then calculated by subtracting W_WF_ from W_WS_. The wet weight measurement method keeps the cells in the sample alive and they continue to grow after the measurement, which makes the experiment more accurate with a smaller number of replicated samples than in the case of using the conventional method. A correlation between W_WB_ and W_DB_ of over 200 samples was developed as an equation to estimate W_DB_ (W_Est_DB_) from W_WB_ (equation 5).

5 $${W}_{Est\_DB}=0.28\cdot {{\rm{W}}}_{WB}-0.065$$

The R^2^ value for the correlation between W_DB_ and W_WB_ was 99.04% (Supplementary Fig. S1). According to the results, the wet weight measurement is deemed a reliable method to accurately estimate the dry weight of the corresponding sample.

The biomass surface density (C) and surface productivity (P) on the cultivation day of n were calculated as follows (Eqs 6 and 7).

6 $${\rm{C}}\,(g/{m}^{2})={W}_{Est\_DB}/A\,$$

7 $${\rm{P}}(g/{m}^{2}/day)=C/n\,$$

where A is the inoculated area of the biofilm (0.0007 m^2^)

### Determination of lipid content and its fatty acid composition

Lipids were measured based on the amount of Fatty Acid Methyl Esters (FAMEs) obtained through transesterification of fatty acids as follows. A sample with a biofilm was harvested by rinsing with distilled water. The collected biomass was then dewatered using centrifugation and lyophilized at −50 °C for 2 days. The obtained dry biomass was ground using a mortar prior to fatty acid analysis through a modified Folch method. About 10 mg of biomass was weighed and placed in a 15 ml glass tube. 2 ml of chloroform-methanol solution (2:1, v/v), following the modified Folch method^37^, along with a known amount of heptadecanoic acid (C17:0) as an internal standard, and sulfuric acid and additional methanol were used for the reaction at 100 °C for 20 min to carry out transesterification. The FAMEs in the organic phase were filtered into a vial and analyzed using a gas chromatograph (HP6890, Agilent, USA) with a flame ionized detector and an HP-INNOWAX column (30 m × 0.32 mm × 0.5 μm, Agilent, USA). The fatty acid composition was identified and quantified by a comparison of the retention times and peak areas of the internal FAME standards.

### Experimental design and optimization by RSM

In order to investigate the relationship between independent and dependent variables, RSM was used with one of the central composite design method, a central composite face-centered (CCF) design. In this study, the independent variables were light intensity and CO_2_ concentration, the two most important factors affecting photoautotrophic cell growth, and the dependent variables were biomass surface productivity and lipid surface productivity. The range of both independent variables were 200 μE/m^2^/s, 500, μE/m^2^/s, and 800 μE/m^2^/s for light intensity, and 0.05% (ambient air), 5%, and 10% for CO_2_ concentration, corresponding to the coded levels of −1, 0, and 1. The coded levels of the independent variables were determined by CCF design, which makes α equal to ± 1. The total number of experimental runs was 29 with 2 blocks based on the calculation of ${{\rm{n}}}_{r}\cdot ({2}^{k}+2k)+{n}_{o}$, where k is the number of independent variables and *n*_*o*_ is the number of experiments repeated at the center point, which was 5 in this study, and n_*r*_ is the number of replicates, which was 3 in this study. The response variables were fitted by following a second-order polynomial equation (equation 8):

8 $$y={\beta }_{0}+\sum _{i}\,{\beta }_{i}{x}_{i}+\sum _{i}\sum _{j}\,{\beta }_{ij}{x}_{i}{x}_{j}+\sum {\beta }_{ii}{x}_{i}^{2}$$

where y is the experimental data of dependent variables, *β*_0_ is the intercept term, *β*_i_, *β*_ij_, and *β*_ii_ are the coefficients or measures of the effects of independent variables of x_i_, x_i_x_j_, and ${{\rm{x}}}_{{\rm{i}}}^{2}$, respectively. In this study, i and j denote light intensity and CO_2_ concentration, respectively. A software program, Design Expert 7.0 (Stat-Ease, Inc., MN, USA), was used for the regression analysis and analysis of variance (ANOVA).

## Electronic supplementary material

Supplementary information

## Data Availability

The datasets generated during and/or analyzed during the current study are available from the corresponding authors on reasonable request.
