# Author Correction: Statistical optimization of light intensity and CO_2_ concentration for lipid production derived from attached cultivation of green microalga *Ettlia* sp.

**DOI:** 10.1038/s41598-019-43507-w

**Published:** 2019-06-05

**Authors:** Sungwhan Kim, Myounghoon Moon, Minsoo Kwak, Bongsoo Lee, Yong Keun Chang

**Affiliations:** 1 Department of Chemical and Biomolecular Engineering, KAIST, 291, Daehak-ro, Yuseong-gu, Daejeon, 34141 Republic of Korea; 2 Advanced Biomass R&D Center, 291, Daehak-ro, Yuseong-gu, Daejeon, 34141 Republic of Korea

Correction to: *Scientific Reports* 10.1038/s41598-018-33793-1, published online 18 October 2018

The original version of this Article contained a typographical error in the spelling of the author Myounghoon Moon, which was incorrectly given as Myunghoon Moon. This has now been corrected in the PDF and HTML versions of the Article, and in the accompanying Supplementary Information file.

