# Supplementary material for: Statistical optimization of light intensity and CO2 concentration for lipid production derived from attached cultivation of green microalga Ettlia sp
Source: Sci Rep. 2018 Oct 18;8:15390. doi: 10.1038/s41598-018-33793-1 (PMC6193934; doi:10.1038/s41598-018-33793-1)

Supplementary Information

Statistical optimization of light intensity and CO_2 concentration for lipid production derived from attached cultivation of *Ettlia* sp.

Sungwhan Kim[a], Myounghoon Moon[b], Minsoo Kwak[a], Bongsoo Lee[a,b*], Yong Keun Chang[a,b*]

[a]*Department of Chemical and Biomolecular Engineering, KAIST, 291, Daehak-ro, Yuseong-gu, Daejeon 34141, Republic of Korea*

[b]*Advanced Biomass R&D Center, 291, Daehak-ro, Yuseong-gu, Daejeon 34141, Republic of Korea*

*Corresponding authors:

Bongsoo Lee

Tel: +82 42 350 5964; Fax: +82 42 860 3910

E-mail address: bongsoolee@kaist.ac.kr

Yong Keun Chang

Tel: +82 42 350 3927; Fax: +82 42 860 3910

E-mail address: changyk@kaist.ac.kr

Table S1. Independent variables and their coded and actual values used for CCF design

Independent variable	Units	Symbol	Coded levels		
			-1	0	1
Light intensity	$\mu E/m^2/s$	A	200	500	800
CO_2 concentration	vol %	B	0.05	5	10

Table S2. Observed and predicted biomass surface productivity of *Ettlia* sp. YC001 on day 4.

Run order	A	B	Biomass surface productivity ($g/m^2/day$) observed	Biomass surface productivity ($g/m^2/day$) predicted
1	1	0	23.01	24.65
2	-1	0	19.87	19.83
3	1	-1	6.99	5.44
4	1	1	23.25	26.54
5	-1	-1	7.08	4.13
6	-1	-1	5.55	4.13
7	0	-1	3.41	6.99
8	0	0	25.72	24.44
9	0	1	26.38	24.59
10	-1	1	18.72	18.22
11	1	1	25.95	26.54
12	0	1	25.52	24.59
13	0	0	23.54	24.44
14	1	1	29.53	26.54
15	-1	0	18.84	19.83
16	0	0	26.07	24.44
17	-1	-1	4.21	4.13
18	-1	1	17.69	18.22
19	1	0	22.77	24.65
20	0	-1	4.44	6.99
21	1	-1	7.13	5.44
22	1	0	26.01	24.65
23	-1	0	16.89	19.83
24	0	1	23.35	24.59
25	0	-1	5.62	6.99
26	0	0	28.40	24.44
27	1	-1	5.80	5.44
28	-1	1	18.23	18.22
29	0	0	25.05	24.44

Predicted data were developed using central composite face-centered (CCF) design used for RSM and observed data of biomass surface productivity were triplicated.

Figure S1. Correlation between weight of wet biomass (W_{WB}) and dry biomass (W_{DB}).

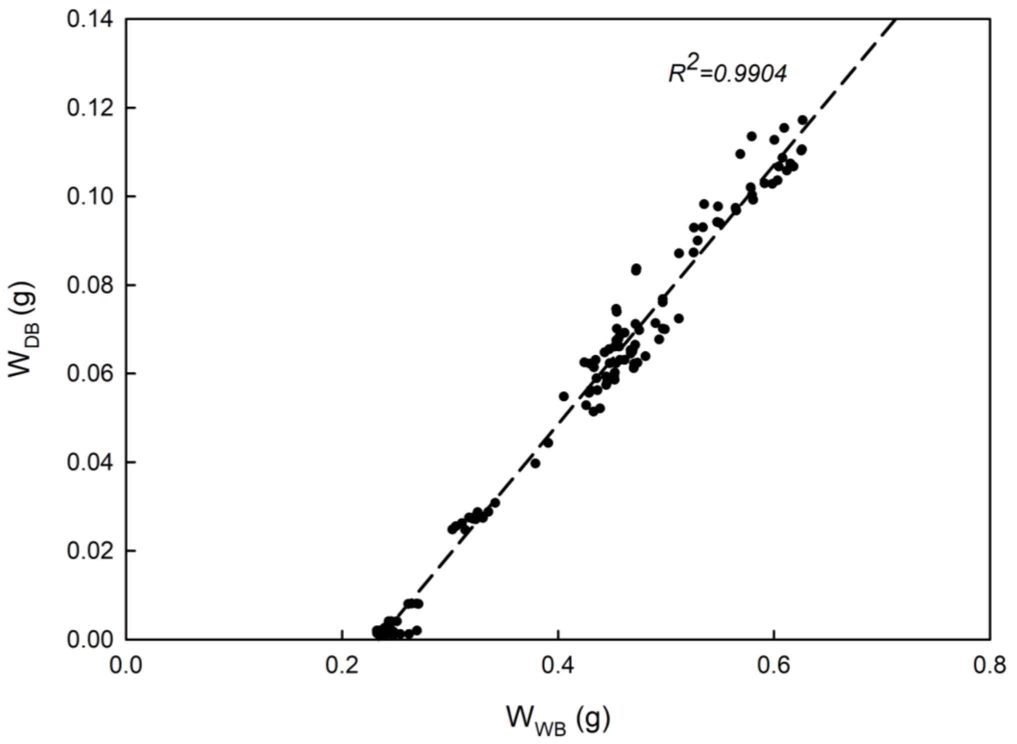

Supplement: Supplementary file 1 — Supplementary information [file 41598_2018_33793_MOESM1_ESM.pdf]
